# Proteasome inhibition enhances the efficacy of volasertib-induced mitotic arrest in AML *in vitro* and prolongs survival *in vivo*

**DOI:** 10.18632/oncotarget.15503

**Published:** 2017-02-18

**Authors:** Dominik Schnerch, Julia Schüler, Marie Follo, Julia Felthaus, Dagmar Wider, Kathrin Klingner, Christine Greil, Justus Duyster, Monika Engelhardt, Ralph Wäsch

**Affiliations:** ^1^ Department of Hematology, Oncology and Stem Cell Transplantation, Medical Center-University of Freiburg, Faculty of Medicine, University of Freiburg, Freiburg, Germany; ^2^ Oncotest GmbH, Freiburg, Germany

**Keywords:** AML, antimitotic therapy, APC/C, mitotic slippage, proteasome inhibition

## Abstract

Elderly and frail patients, diagnosed with acute myeloid leukemia (AML) and ineligible to undergo intensive treatment, have a dismal prognosis. The small molecule inhibitor volasertib induces a mitotic block via inhibition of polo-like kinase 1 and has shown remarkable anti-leukemic activity when combined with low-dose cytarabine. We have demonstrated that AML cells are highly vulnerable to cell death in mitosis yet manage to escape a mitotic block through mitotic slippage by sustained proteasome-dependent slow degradation of cyclin B. Therefore, we tested whether interfering with mitotic slippage through proteasome inhibition arrests and kills AML cells more efficiently during mitosis. We show that therapeutic doses of bortezomib block the slow degradation of cyclin B during a volasertib-induced mitotic arrest in AML cell lines and patient-derived primary AML cells. In a xenotransplant mouse model of human AML, mice receiving volasertib in combination with bortezomib showed superior disease control compared to mice receiving volasertib alone, highlighting the potential therapeutic impact of this drug combination.

## INTRODUCTION

As a result of continuous therapeutic advances in recent decades, the prognosis of acute myeloid leukemia (AML) has improved for young and healthy individuals eligible for intensive therapy [1, 2]. In contrast, in most cases elderly and more unfit patients diagnosed with AML still face a dismal prognosis and often succumb to their disease shortly after the time of diagnosis [2–4]. The use of low-dose cytarabine [5]. hydroxyurea [5]. azacitidine [6]. decitabine [7] or clofarabine [8] was shown to slow down the progressive course of AML in frail patients. More intensive approaches, which could induce more sustained remissions even in elderly patients, are often limited by the occurrence of life-threatening adverse events [9].

Small molecule inhibitors are becoming more and more important due to a remarkable power in disease control, comparably low side effects and good tolerability [10, 11]. The latter characteristics hence placed these agents into focus for use in elderly and frail patients, ineligible to undergo intensive treatment and suffering from aggressive diseases, such as AML. Targeting polo-like kinase 1 (Plk1) is an efficient antimitotic treatment approach [12] with BI6727 (volasertib) demonstrating a striking effect in AML when combined with low-dose cytarabine [13]. While antimitotic drugs play only a negligible role in the treatment of myeloid neoplasias, volasertib, when combined with low-dose cytarabine, induced significantly better clinical outcomes in patients suffering from AML as compared to patients receiving low-dose cytarabine alone [13].

Mechanistically, antimitotic drugs induce a mitotic delay which triggers induction of apoptosis during mitosis [14]. Regular mitotic exit requires activity of the anaphase-promoting complex (APC/C) in order to allow transition into G1 phase [15–18]. Specifically, proper alignment of all chromosomes at the metaphase plate relieves APC/C inhibition which then triggers the rapid degradation of mitotic B-type cyclins by the ubiquitin-proteasome system to promote mitotic exit [19]. Since antimitotic drugs perturb the proper line-up and attachment of chromosomes at the metaphase plate, continued APC/C inhibition and, in turn, a high abundance of mitotic cyclin B arrest cells in mitosis [14]. However, cancer cells find ways to escape from mitosis and get out of this vulnerable stage. For example, in the presence of inhibited APC/C, cells can still exhibit slow but permanent degradation of cyclin B [20]. This allows the cells to slip out of mitosis once cyclin B levels have fallen below a critical level, which is insufficient to maintain mitotic arrest. It has been observed that slow cyclin B degradation depends on the proteasome and proteasome inhibition blocked slow cyclin B degradation leading to robust mitotic arrest [20, 21].

Here we address the question of whether slow cyclin B degradation, the main driver of mitotic slippage, can be targeted by proteasome inhibition to consolidate a mitotic block for AML therapy. Using a combination of live-cell imaging at the single cell level and fluorescence-activated cell sorting, we demonstrate that interference with slow cyclin B degradation is feasible with therapeutic proteasome inhibitor concentrations. We focused on the combination of volasertib with the proteasome inhibitor bortezomib to consolidate a mitotic block. Finally, we took advantage of a xenotransplant mouse model of human-derived AML to test the feasibility of this combination *in vivo* and provided evidence that the combined use of volasertib and bortezomib may lead to superior disease control and longer survival.

## RESULTS

### APC/C^Cdh1^ and APC/C^Cdc20^ support slow cyclin B degradation during a mitotic block in U2Os cells

How slow cyclin B degradation is mediated in spindle assembly checkpoint (SAC)-arrested cells with sequestered Cdc20 remains incompletely resolved to date. There are reports arguing that this may be APC/C-dependent [22].

**Figure 1:**
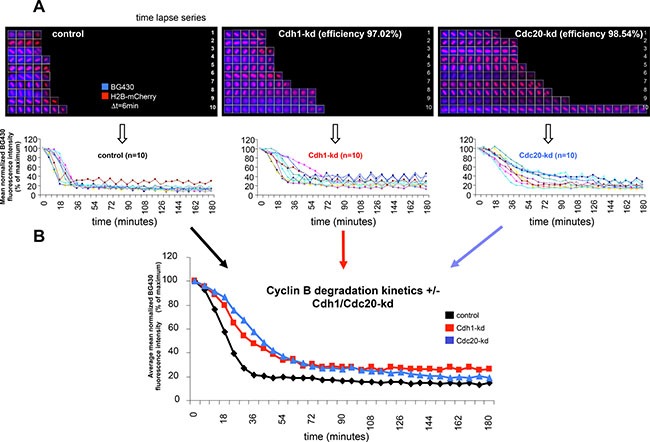
Effect of Cdh1- and Cdc20-kd on cyclin B degradation kinetics in unperturbed single cells (**A**) Unperturbed BG430-stained CYS cells were followed by live-cell imaging at the single cell level. Time-lapse series depict chromatin (in red) and abundance of BG430-bound cyclin B-YFP-SNAP protein (in blue) in individual CYS cells during mitosis (see upper panels). Cyclin B-YFP-SNAP degradation kinetics as derived from the shown *n* = 10 individual CYS cells are presented in the diagrams below the time-lapse series. Time-lapse series and degradation curves to the left are derived from control cells. Time-lapse series and degradation curves in the center are derived from CYS cells following Cdh1-kd with a calculated kd efficiency of 97.02%. Time-lapse series and degradation curves to the right are derived from CYS cells following Cdc20-kd with a calculated kd efficiency of 98.54%. (**B**) The diagram shows averaged cyclin B degradation curves (data are derived from the cells shown in (A)) of control cells (black line), Cdh1-kd cells (red line) and Cdc20-kd cells (blue line).

To further address this question, we analyzed the effects of Cdh1- and Cdc20-knockdown (kd) on cyclin B degradation using a SNAP-reporter system in unperturbed single U2Os cells undergoing mitosis as described previously [21]. To this end, we followed individual cells by time-lapse microscopy. A set of ten time-lapse series, representative of the tested conditions, is shown and the derived BG430 fluorescence intensity traces, which indicate the stability of cyclin B, are provided below (Figure 1A). The averaged mean fluorescence intensity curves indicate that Cdh1-kd and Cdc20-kd both reduce the extent of cyclin B degradation during mitotic exit (Figure 1B). In agreement with existing evidence [23]. Cdc20-kd had a stronger effect in mitosis while Cdh1-kd cells exhibited higher levels of cyclin B later during G1 phase. In addition, we also observed an effect of Cdh1-kd on cyclin B earlier in mitosis (Figure 1B).

**Figure 2:**
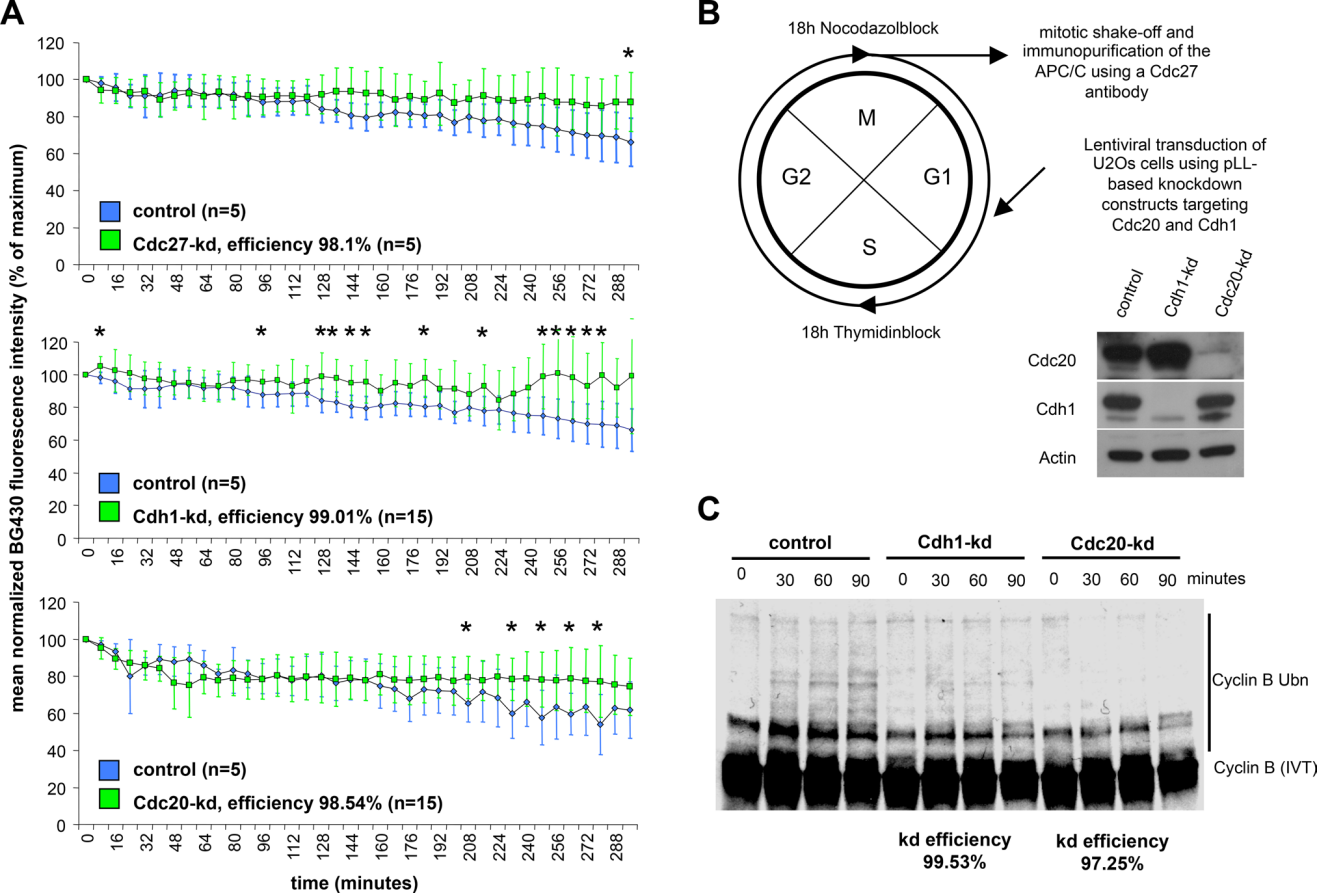
Slow degradation of cyclin B during a mitotic block is influenced by the activating APC/C subunits Cdc20 and Cdh1 (**A**) Cyclin B-YFP-SNAP degradation kinetics in U2Os cells during a nocodazole-induced mitotic block are shown. Degradation kinetics at the single cell level in the presence of a Cdc27-kd (squares filled in green) compared to control cells (rhombs filled in blue) are shown in the upper diagram. Degradation kinetics in the center reveal the effect of a Cdh1 knockdown (squares filled in green) while degradation kinetics in the lower diagram show the difference between Cdc20-kd cells (green squares) and control cells (blue diamonds). Calculated kd efficiencies are listed in the lower right part of each diagram. The control degradation curves in the upper and center diagram are identical since the data for the Cdc27-kd-, Cdh1-kd- and control curves are derived from the same experiment. * indicates *p* < 0.05 at the indicated time point. (**B**) The scheme in the upper left indicates the processing of cells prior to harvest for the *in vitro* ubiquitination assay. Western Blot below the scheme indicates the efficacy of the Cdh1- and Cdc20-kd. (**C**) Autoradiogram to the lower right shows the abundance of ubiquitylated cyclin B at the indicated time points following incubation with APC/C, which was immunopurified from synchronized kd and control cells during mitotic block. Calculated kd efficiencies (based on the Western Blot above) are shown below the autoradiogram.

We next analyzed the kinetics of slow cyclin B degradation during mitotic block. Therefore, we studied the effects of Cdh1-kd and Cdc20-kd on slow cyclin B degradation in cells, which were treated with spindle disruptive doses of nocodazole. Direct interference with the APC/C by kd of the Cdc27 core subunit served as a positive control. All three kds reduced the extent of slow cyclin B degradation during mitotic block (Figure 2A). These results confirmed that Cdh1 and Cdc20 are both involved in slow cyclin B degradation during mitosis and, in consequence, support mitotic slippage.

To further define the relative contributions of Cdh1 and Cdc20 to slow cyclin B degradation, we immunopurified APC/C from extracts of Cdh1- and Cdc20-kd cells which were arrested in a nocodazole block and measured the extent of cyclin B ubiquitinylation. A scheme for the experimental procedure of cell harvest and WB indicating kd efficiency is shown in Figure 2B. While Cdh1-kd reduced cyclin B ubiquitinylation in mitosis, Cdc20-kd strongly diminished cyclin B ubiquitinylation. These results demonstrate that both forms of the APC/C, APC/C^Cdh1^ and APC/C^Cdc20^, are active in prometaphase, with Cdc20 exerting a stronger effect than Cdh1 (Figure 2C).

### Therapeutic doses of the proteasome inhibitor bortezomib reduce slow cyclin B degradation during mitotic block in U2Os cells

Next we asked whether low doses of proteasome inhibitors, at amounts resembling therapeutic levels in patient serum (Supplementary Figure 1) [24], are sufficient to interfere with slow cyclin B degradation.

**Figure 3:**
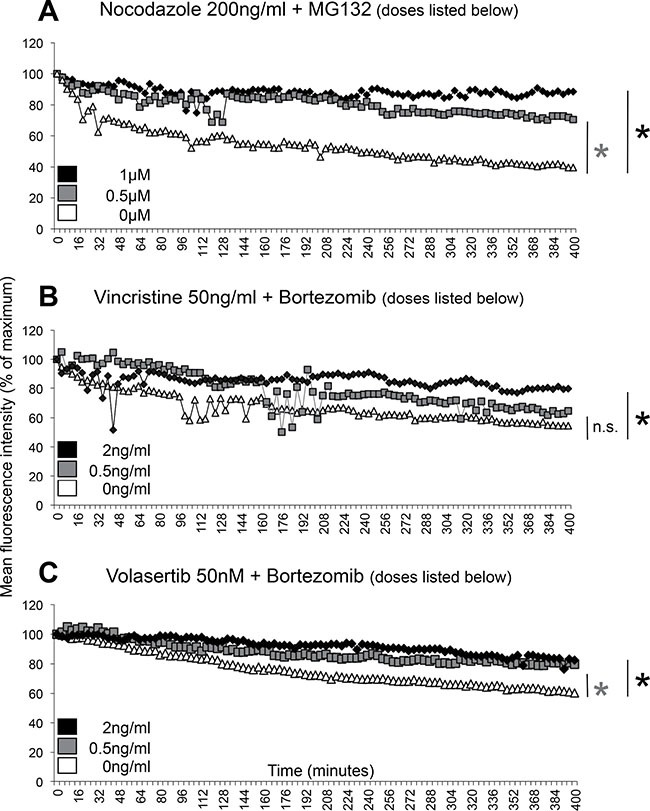
Therapeutic doses of bortezomib reduces slow cyclin B degradation during a mitotic block Cyclin B degradation slopes as determined by live-cell imaging of individual U2Os cyclin B-SNAP clone 11 cells during a mitotic block are shown. (**A**) U2Os cyclin B-SNAP reporter cells were treated with 200 ng/ml nocodazole to induce a stable mitotic block and MG132 at 0 μM (white triangles), 0.5 μM (grey squares) and 1 μM (black diamonds). (**B**) U2Os cyclin B-SNAP reporter cells were treated with 50 ng/ml vincristine to induce a stable mitotic block and bortezomib at 0 ng/ml (white triangles), 0.5 ng/ml (grey squares) and 2 ng/ml (black diamonds). (**C**) U2Os cyclin B-SNAP reporter cells were treated with 50 nM volasertib to induce a stable mitotic block and bortezomib at 0 ng/ml (white triangles), 0.5 ng/ml (grey squares) and 2 ng/ml (black diamonds). Degradation slopes represent mean values derived from 3 individual representative cells undergoing a mitotic block. *p < 0.05, at *t* = 400 min.

First, we tested the effect of the experimental proteasome inhibitor MG132 on cyclin B-SNAP-expressing U2Os cells (clone 11 as described in [21], which were arrested in mitosis as a consequence of spindle disruption caused by high doses of the microtubule-destabilizing agent nocodazole. We observed that low doses of MG132 (0.5 μM) were sufficient to induce a decrease in slow cyclin B degradation, while MG132 at a dose of 1 μM was able to completely abolish slow cyclin B degradation and mimic the degradation curve which was seen following application of high dose MG132 (10 μM) in our previous report (Figure 3A) [21].

Both vincristine, a microtubule-destabilizing agent, and bortezomib, a proteasome inhibitor, are frequently used in the clinic in therapeutic regimens to treat a wide variety of cancers. Therefore, in the next experiment we combined vincristine (applied at high doses of 50 ng/ml to induce a stable mitotic block) and bortezomib. Serum concentrations of bortezomib after application of therapeutic doses usually range between 0.5 and 2 ng/ml for up to 72 hours [24, 25]. Thus, we studied cyclin B degradation kinetics in vincristine-arrested cells in the presence of 0.5 ng/ml and 2 ng/ml bortezomib. In agreement with our results seen in nocodazole and MG132-treated cells, slow cyclin B degradation was also reduced when vincristine and bortezomib were combined with the effect significant when 2 ng/ml of bortezomib was used (Figure 3B).

Since we also observed cyclin B slow degradation during a mitotic delay when we applied the Plk1-inhibitior volasertib (Supplementary Figure 2), we next combined volasertib and bortezomib. Again, cyclin B slow degradation was significantly reduced upon proteasome inhibition using bortezomib at a concentration of 0.5 and 2 ng/ml in volasertib-treated cells undergoing mitotic delay (Figure 3C).

Taken together, these data provide evidence that the combined use of therapeutic doses of the proteasome inhibitor bortezomib during antimitotic treatment blocks slow cyclin B degradation *in vitro* to support a robust mitotic arrest.

### Therapeutic doses of proteasome inhibitor prolong a mitotic delay in myeloblastic Kasumi-1 cells

We recently reported that the AML cell line Kasumi-1 underwent only a transient mitotic delay upon treatment with spindle poison [26]. In light of this inherent limitation in inducing a robust mitotic block, we considered Kasumi-1 cells to be an ideal tool to test the combination of an antimitotic agent with a proteasome inhibitor. Moreover, bortezomib has already shown considerable antileukemic activity, especially in myelomonocytic and FLT3-mutant leukemias [27–29].

**Figure 4:**
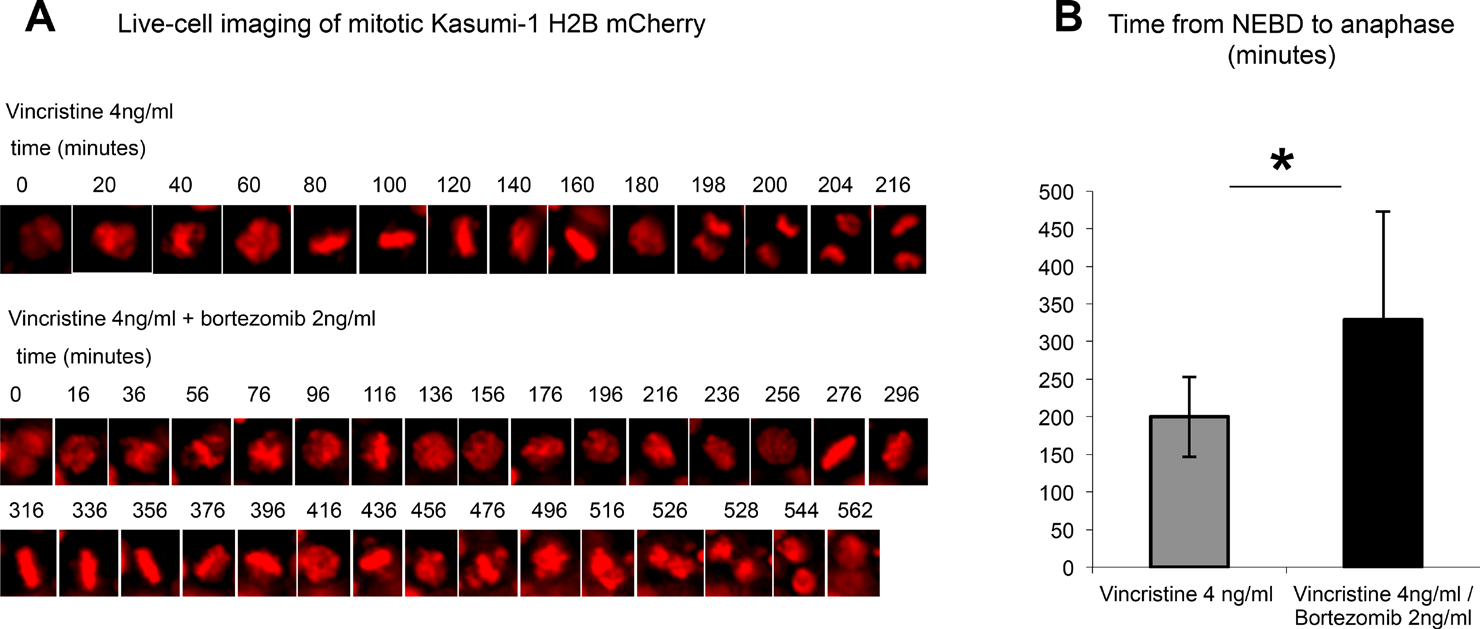
Concomitant application of bortezomib prolongs a vincristine-induced mitotic delay in Kasumi-1 cells (**A**) Time series of representative H2B-mCherry-expressing Kasumi-1 cells studied by live-cell imaging during a mitotic delay in the presence of vincristine (upper time-lapse series) and vincristine combined with bortezomib (lower time-lapse series). (**B**) Quantification of the time intervals from NEBD until anaphase in individual Kasumi-1 cells (*n* = 13) undergoing treatment with vincristine or vincristine combined with bortezomib. *p < 0.05.

To this end, Kasumi-1 cells stably expressing H2B-mCherry as a chromatin marker were followed via live-cell imaging and the time between nuclear envelope breakdown (NEBD) and chromosomal segregation was quantified. When challenged with 4 ng/ml vincristine, a concentration seen in patient serum following application of a therapeutic dose of 2 mg i.v. [30], Kasumi-1 cells exhibited a mitotic delay, yet still managed to segregate their chromosomes (Figure 4A, upper panel). This mitotic delay was significantly prolonged by approximately 1.5-fold when we used vincristine in combination with the proteasome inhibitor bortezomib at the therapeutic dose of 2 ng/ml (Figure 4A lower panel and Figure 4B).

### Therapeutic doses of proteasome inhibitor increased the efficacy of antimitotic treatments in Kasumi-1 cells and primary patient-derived AML blasts

**Figure 5:**
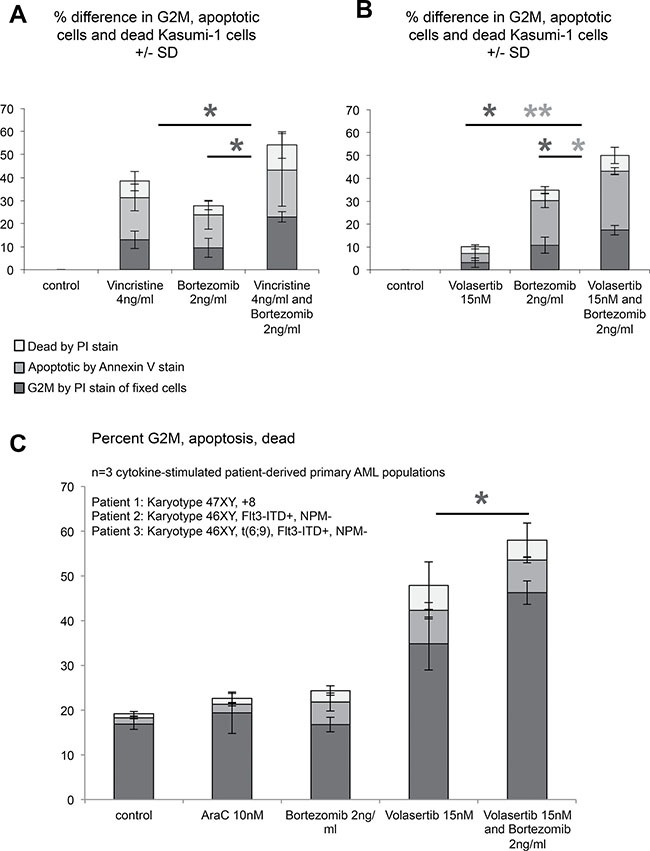
Kasumi-1 and primary patient-derived AML cells respond more efficiently to antimitotic therapy upon addition of bortezomib (**A**, **B**) Response of Kasumi-1 cells to antimitotic therapy, proteasome inhibition and antimitotic therapy with concomitant proteasome inhibition was assessed by flow cytometry. Percentage of increase in G2/M phase (dark grey), apoptosis (light grey) and necrotic cells (white) as compared to controls. Values shown represent the mean +/− standard deviation as determined from *n* = 3 independent experiments. (**C**) Response of cytokine-stimulated primary patient-derived AML populations to low dose cytarabine, volasertib, bortezomib and volasertib with concomitant application of bortezomib was assessed by flow cytometry. Shown data are derived from primary cell populations from *n* = 3 different AML patients. Biological characteristics are listed in the upper part of the histogram. Data shown represent the mean of absolute percentages +/− standard deviation. Color coding of the bars was done as described in A, B.*p < 0.05, **p < 0.01.

To assess the response of AML cells to the combination of an antimitotic agent combined with a proteasome inhibitor, we studied the extent to which Kasumi-1 and primary patient-derived AML cells underwent a G2/M block (dark grey) or cell death (apoptosis - intermediate grey, necrosis -light grey) (Figure 5A).

We noted that the combined use of vincristine and bortezomib at therapeutic doses significantly increased the percentage of cells in the G2/M fraction of the cell cycle (Figure 5A). When we used volasertib and bortezomib in combination, not only was the accumulation of cells in G2/M significantly enhanced, but also the fraction of cells undergoing apoptosis (Figure 5B). Of note, an enhanced accumulation in mitosis was also observed when the antimitotic agent nocodazole was combined with low doses of the proteasome inhibitor MG132 (Supplementary Figure 3A), confirming that our observations are not bortezomib-specific effects, but also reproducible with different proteasome inhibitors.

We next asked the question, whether a further increase of the doses of proteasome inhibitors led to more profound mitotic delay in Kasumi-1 cells. Quite interestingly, the accumulation of Kasumi-1 cells in G2M upon exposure to higher doses of MG132 and bortezomib was lower in nocodazole-, vincristine- and volasertib-treated cells (Supplementary Figure 3A–3C) lending support to the hypothesis that a higher number of cells was arrested before entering mitosis.

To test the combined use of antimitotic agents and a proteasome inhibitor at therapeutic doses in primary patient-derived AML cells, we stimulated primary AML blasts from three different patients with cytokines to grow them in culture, as recently described [26]. Cells were pre-cultured for 72 hours and challenged with the same treatment combinations as Kasumi-1 cells for 24 hours. Treatment with volasertib already had a marked effect on G2/M arrest (Figure 5C). Importantly, however, combining volasertib with 2 ng/ml bortezomib led to a further increase in the proportion of cells accumulating in G2/M (Figure 5C). Since overexpression of cyclin B directly boosted the accumulation of volasertib-treated cells in G2/M, bortezomib-induced stabilization of cyclin B may cause the increased accumulation in G2/M (Supplementary Figure 4). In contrast, 10nM cytarabine, mimicking concentrations seen with low-dose cytarabine treatment, and bortezomib, given as a monotherapy, only induced marginal effects on accumulation in G2/M (Figure 5C).

### Overexpression but not bortezomib-induced stabilization of Mcl-1 reduced the efficacy of antimitotic treatment and the extent of leukemia cell death

We recently demonstrated that expression levels of the antiapoptotic regulator Mcl-1 are significantly decreased in AML cell lines as compared to lymphoblastic cell lines [26]. A reduction in Mcl-1 expression levels by targeted degradation is a trigger of cell death during mitotic block [31]. Therefore, we asked whether low Mcl-1 expression levels in AML cells make these cells *a priori* more vulnerable to antimitotic treatment when the application of an antimitotic agent and a proteasome inhibitor are combined. Since Mcl1 abundance in mitotic cells is controlled by proteasome-dependent degradation, treatment with a proteasome inhibitor might also lead to stabilization of Mcl1 protein levels. This could in turn lead to resistance to cell death during mitosis in AML cells.

**Figure 6:**
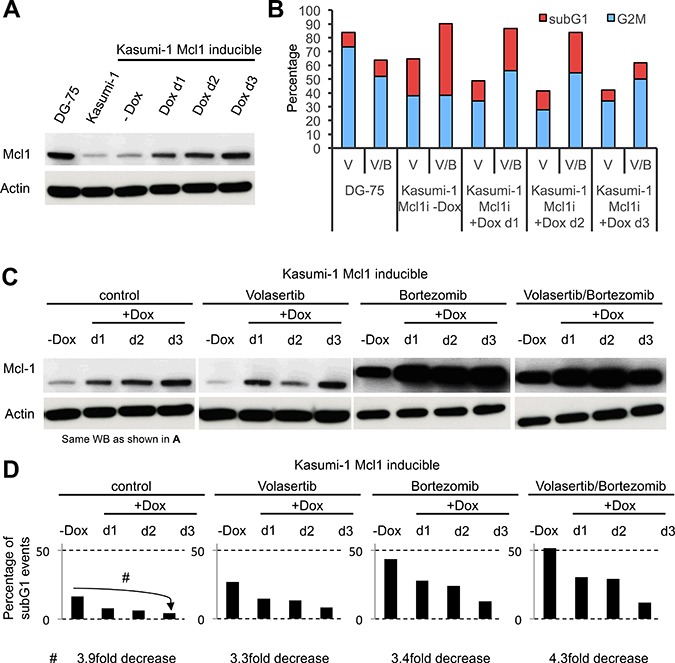
Bortezomib-induced stabilization of Mcl-1 does not protect Kasumi-1 cells from therapy-induced cell death (**A**) Western Blot illustrating the protein expression levels of Mcl1 in lymphoblastic DG-75 cells, myeloblastic Kasumi-1 cells and Mcl1-inducible Kasumi-1 cells +/− induction of Mcl1 expression by doxycycline. (**B**) Diagram indicating the percentage of cells undergoing mitotic delay (G2/M phase; blue) and dead cells (subG1; red) in DG75 cells and Mcl1-inducible Kasumi-1 cells. (**C**) Western Blot illustrating the expression levels of Mcl1 in Mcl-1-inducible Kasumi-1 cells. Note that the Western Blot to the left is the same one as shown in (A), except that the bands for DG-75 and Kasumi-1 wild type cells are not shown. (**D**) Diagrams illustrate the extent of cell death of Mcl1-inducible Kasumi-1 cells as determined by the percentage of subG1 events upon induction of Mcl1 overexpression for the indicated times and in the presence of the indicated drug treatments.

To address this question we engineered a Kasumi-1 cell line which allowed overexpression of Mcl1 following induction by doxycycline. Levels of Mcl1 overexpression, achieved in Kasumi-1 cells after three days of induction, were reminiscent of the Mcl1 expression levels seen in lymphoblastic DG75 cells (Figure 6A). Remarkably, overexpression of Mcl1 caused an approximately 4-fold decrease in the percentage of subG1 cells at day 3 under all tested treatment conditions (Figure 6A–6D). In contrast, the higher Mcl-1 expression levels observed following 24 hours of proteasome inhibition (Figure 6C) were poor predictors of cell fate. While Kasumi-1 cells treated with bortezomib showed a profound stabilization of Mcl1 expression levels, the subG1 fractions were even higher (Figure 6B and 6D). However, the subG1 fraction decreased in a time-dependent manner upon Mcl1 overexpression, both with and without bortezomib (Figure 6A–6D).

### Combined administration of a proteasome inhibitor with volasertib leads to prolonged survival *in vivo*

We then analyzed whether the combination of bortezomib and volasertib also improves the response *in vivo*. In these experiments, Kasumi-1 cells were transplanted into recipient mice. Following engraftment and development of overt leukemic disease, the mice were randomized to receive either bortezomib or volasertib alone, the combination of bortezomib and volasertib, or no treatment.

**Figure 7:**
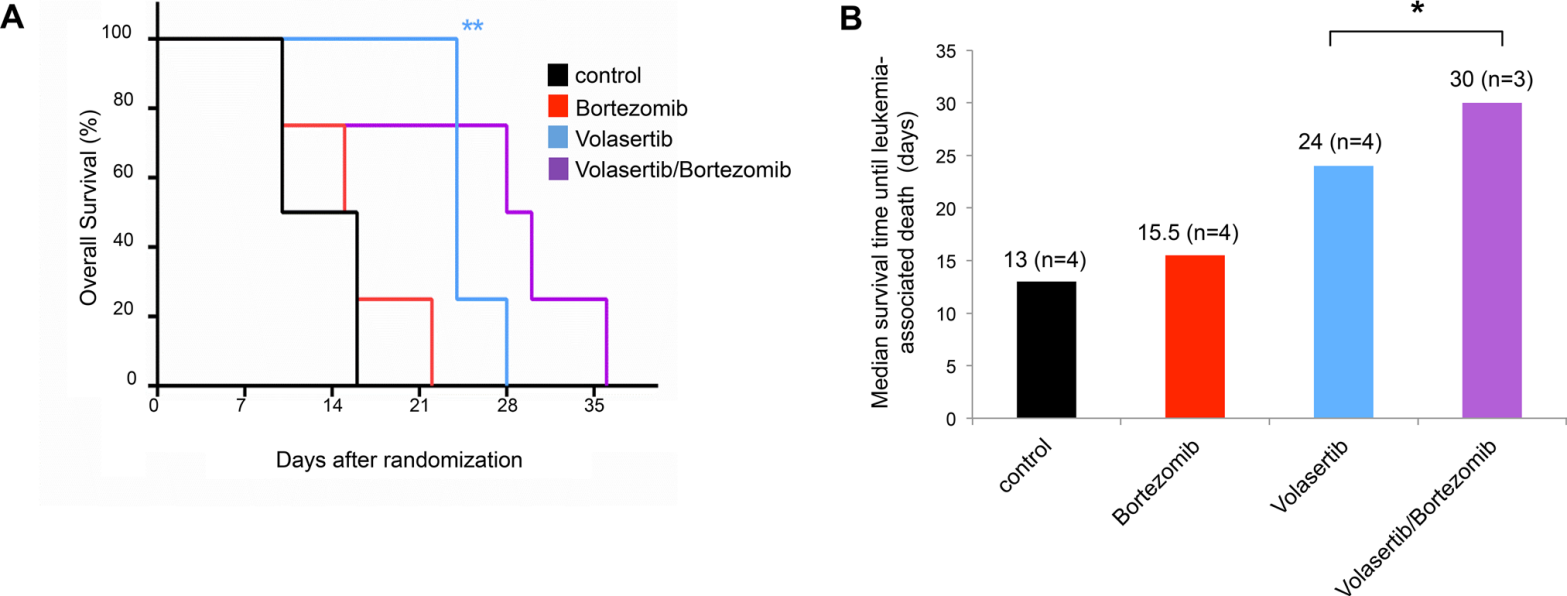
Bortezomib in combination with volasertib induces improved disease control in a xenotransplant mouse model of human AML (**A**) Kaplan Meier curves as determined for xenotransplant mice bearing human Kasumi-1-derived AML upon treatment as indicated in the upper right. *n* = 4 mice per cohort. (**B**) Diagram shows median survival time until leukemia-associated mortality. *p < 0.05, **p < 0.01.

While mice in the control and in the bortezomib arms died early due to progressive disease (median survival 13 and 15.5 days, respectively), mice receiving volasertib monotherapy exhibited longer survival (median survival 24 days) (Figure 7A and 7B). However, the mice treated with the bortezomib/volasertib combination had the longest survival times: Of note, one mouse in the combination group died as early as the untreated mice for unknown reasons (when excluding the early death median survival was 30 days). In further support of these results, we also noted an improved disease control as determined by the abundance of leukemia cells in the peripheral blood in a pioneering experiment done in a Molm13-based xenotransplant model (Supplementary Figure 5).

## DISCUSSION

Targeting mitosis is an effective therapeutic approach for a wide variety of cancers. While classic antimitotic drugs, such as vinca alkaloids and taxanes, have proven their profound antimitotic activity for decades, a new generation of antimitotic drugs has been developed in recent years. These small molecule inhibitors target key enzymes which are critical for mitotic progression, such as polo-like kinase or kinesin motor proteins. These inhibitors induce a strong mitotic delay, often with lower side effects than the classic drugs. However, mitotic slippage, which is the main limiting factor in antimitotic therapy, allowing cells to escape from a mitotic block, persists. In this study we addressed the question as to whether slow cyclin B degradation, the driver of mitotic slippage, can be targeted by proteasome inhibition to consolidate a mitotic block for AML therapy using therapeutic doses.

We observed additive effects when an antimitotic drug was combined with a proteasome inhibitor. The most striking effects were seen when the Plk1-inhibitor volasertib was combined with the proteasome inhibitor bortezomib. These additive effects were not restricted to cell lines but were also seen in primary patient-derived AML cells during cytokine-driven expansion in tissue culture. In an *in vivo* setting, the combined use of volasertib and bortezomib led to superior disease control and longer survival before leukemia-associated death compared to administration of volasertib alone.

A prerequisite for antimitotic therapy to work efficiently is that cells have to progress to mitosis where they are then supposed to die. However, most cell cycle regulators are targeted for degradation by the proteasome. For this reason, we considered the possibility that proteasome inhibition might also block cells at checkpoints outside of mitosis. While we cannot exclude that low fractions of cells are arrested at checkpoints in the G1-, S- and G2-phases, there are several arguments which led us to conclude that the administration of low dose bortezomib (at 2 ng/ml) could boost the accumulation of cells primarily in mitosis:

First, Kasumi-1 cells treated with a combination of volasertib and bortezomib were arrested for a longer time in mitosis compared to cells that were treated with volasertib alone. Notably, different reports have shown that bortezomib leads to G2/M arrest and synergistic effects with mitotic inhibitors in myeloid cells [32, 33]. However, we demonstrate here that bortezomib indeed acts during mitosis by using live-cell imaging. In this way, we were able to monitor chromatin behavior during mitosis in Kasumi-1 cells expressing fluorescent protein-tagged histones and measured the time spent in mitosis, and observed a striking prolongation in the length of time between chromatin condensation and anaphase onset.

Second, we demonstrated that therapeutic doses of bortezomib (2 ng/ml) are sufficient to block slow cyclin B degradation.

Third, we noted that volasertib induced a more profound mitotic delay when we counteracted slow cyclin B degradation by induced overexpression of cyclin B. Consistently, a longer mitotic delay upon overexpression of cyclin B has also been seen by others, [34] while the fact that we observe similar responses in volasertib-treated cells upon addition of bortezomib as compared to the overexpression of cyclin B provides strong arguments to claim that the combination of volasertib and bortezomib blocks cells more efficiently in mitosis due to interference with the mitotic degradation of cyclin B.

Moreover, we emphasize that the most striking additive effects were seen with low doses of bortezomib. Higher doses of bortezomib downsized the additive effects, lending support to the notion that a relevant number of cells became arrested at checkpoint before mitosis. Higher doses of bortezomib might therefore protect cells from the collaborative action of both drugs in mitosis. Importantly, this finding represents a nice proof of principle that, in particular situations, lower drug concentrations exert more powerful effects while saving dose-dependent side effects.

Mcl1 is a prominent antiapoptotic factor in mitosis and shows reduced expression in AML [26]. Since expression levels of Mcl1 are regulated by proteasome-dependent degradation [31], we considered the possibility that bortezomib-induced stabilization of Mcl-1 might interfere with induction of cell death during antimitotic therapy in AML. However, bortezomib has been shown to induce caspase-dependent cleavage of Mcl1, which turns wild-type Mcl1 into a Mcl1-derivative with proapoptotic function [35]. Following bortezomib treatment we could indeed observe a faint band below that of wild-type Mcl1. However, due to the low overall abundance of wild-type Mcl1 in Kasumi-1 cells, this faint band only became apparent when cells overexpressing excessive amounts of wild-type Mcl-1 were treated with bortezomib. A bortezomib-specific conversion of Mcl-1 could explain our observation that bortezomib-induced stabilization had no apparent negative influence on therapeutic efficacy. Overexpression of wild-type antiapoptotic Mcl1, instead, caused a profound decrease in the number of dead cells over time. Our data therefore favor the interpretation that bortezomib-induced stabilization of antiapoptotic Mcl1 is counteracted by a concomitant (also bortezomib-induced) modification into a proapoptotic derivative, which keeps AML cells susceptible to therapy.

The combination of bortezomib with antimitotic agents offers a promising perspective to increase the efficacy of antimitotic therapy by targeting mitotic slippage. However, killing cancer cells more efficiently only pays off if the treatment spares healthy cells of an organism in order to allow recovery from disease. Moreover, combining agents might result in an amplification of specific side effects. Bortezomib is routinely used in the treatment of multiple myeloma, and peripheral neuropathy is a dose-limiting toxicity in bortezomib treatment [36]. Since vincristine is known to cause significant neuropathy [37], this drug is not an appropriate candidate for combination with bortezomib. Major side effects seen in patients treated with volasertib were reversible hematological toxicities such as anemia and neutropenia [38], while neuropathy was not seen. Therefore, volasertib is a suitable combination partner for bortezomib.

In our xenotransplant mouse model of human AML, we observed that mice treated with a combination of volasertib and bortezomib, showed longer survival before leukemia-associated death. However, one animal died fairly early during therapy, raising the possibility that it might have succumbed to an enhanced early toxicity of the drug combination. Since all animals in the volasertib mono arm survived the period of treatment, we consider it unlikely that the animal succumbed to a fulminant progression of the transplanted AML. The three remaining animals in the combination arm survived until days 28, 31 and 36, while all animals in the volasertib mono arm died at or before day 28.

In summary, based on the spectra of side effects, volasertib and bortezomib are promising combination partners, and the combined use of both agents at adequate doses shows superior disease control in AML *in vitro* and *in vivo*.

## MATERIALS AND METHODS

### Generation of expression constructs

The double-chimeric fusion protein pLNCX2 cyclin B mut5 YFP SNAP was established based on the pLNCX2 Cyclin B mut5 SNAP construct as described elsewhere [39]. pMXs H2B mCherry IRES Blasticidin was based on pH2B mCherry IRES neo3 (kindly provided by Daniel Gerlich). H2B mCherry was PCR-amplified using AAAAAAAGATCTGCCACCA TGCCAGAGCCAGCGAAGTC as a sense primer and AAAAAACTCGAGTTACTTGTACAGCTCGTCCA as a reverse primer with pH2B mCherry IRES puro2 as a template. The PCR product was processed using a BglII/XhoI digest and introduced into the linearized pMXs IRES Blasticidin backbone (Cell Biolabs), which was linearized using a BamHI/XhoI digest.

For inducible overexpression of Mcl-1 the coding sequence was PCR-amplified from Origene cDNA clone SC315538 (NM_021960) using AAAAAAGGATCCATG TTTGGCCTCAAAAGAAACG as a sense primer and AAAAAAACGCGTCTATCTTATTAGATATGCCAAAC as a reverse primer. The PCR product was processed using a NotI/MluI digest and introduced into the NotI/MluI-linearized pRetroX tightPur backbone (Clontech). Kasumi-1 cells allowing inducible overexpression of Mcl-1 were established as described previously [26].

### Lentivirus-based knockdown plasmids

For knockdown against Cdh1 we took advantage of the target sequence GGATTAACGAGAATGAGAA. For knockdown against Cdc20 we took advantage of the target sequences GCAGAAACGGCTTCGAAAT and AGCAGCAGAAACGGCTTCG. Oligonucleotides containing the target sequence, the complementary sequence, a loop and linker sequences, were designed as previously described [40] and ligated into pLentiLox 3.7 (pLL).

### Lenti- and retroviral transduction

For the generation of lentivirus-containing cell supernatant, 293T cells were transfected with the pLL knockdown vector and packaging plasmids (pMDLg/pRRE, pRSV-Rev, pMD.G). 293T cells were incubated for 10 hours in the presence of calcium phosphate-precipitated plasmid DNA and 20 μM chloroquine. Virus-containing supernatant was collected after 24 and 48 hours. For transduction U2Os cells were incubated in 4 ml of virus-containing supernatant supplemented with 2 ml of fresh normal growth medium and 5 μg/ml hexadimethrine bromide. For generation of retrovirus-containing cell supernatant Phoenix ampho cells were transfected with calcium phosphate-precipitated plasmid DNA in the presence of 20 μM chloroquine. All of the subsequent procedures were conducted as described for the lentiviral transduction.

### Cell culture and antibiotic selection

U2Os cells were a gift from Martin Trepel (Augsburg). U2Os cells expressing Histone H2B-mCherry and the double chimeric fusion protein cyclin B mut5 YFP SNAP (CYS cells) were grown in complete DMEM medium (supplemented with 10% FCS, penicillin/streptomycin, sodium pyruvate, L-glutamine) in the presence of 10 μg/ml blasticidin and 800 μg/ml geneticin as previously described [39]. Monoclonal clone 11 cells were described elsewhere [21] and grown in complete DMEM medium in the presence of puromycin 0.5 μg/ml, geneticin 800μg/ml and blasticidin 30 μg/ml.

For titration experiments addressing the biological effects of different concentrations of antimitotic agents and proteasome inhibitors, U2Os cells were seeded onto 6-well plates at a concentration of 0.5 × 10^6^ per well and grown for 24 h before challenge with substances at different concentrations.

Kasumi-1 cells (a gift from Michael Lübbert, Freiburg) were grown in complete RPMI1640 medium supplemented with 20% FCS, penicillin/streptomycin, sodium pyruvate, and L-glutamine. Mcl-1-inducible Kasumi-1 cells were grown in the above-mentioned complete medium in the presence of 2 μg/ml puromycin and 2400 μg/ml geneticin (as described elsewhere [26]). Kasumi-1 cells expressing H2B mCherry were grown in the presence of 5 μg/ml blasticidin.

The sensitivity of Kasumi-1 cells was tested on 6-well plates. Kasumi-1 cells were seeded at a concentration of 1.2 × 10^6^ per well in 4 ml complete RPMI growth medium and grown for the indicated times under different conditions.

Primary AML cells were isolated after written informed consent from patients presenting with diagnosis of AML at Freiburg University Medical Center. Primary AML cells were grown in RPMI medium supplemented with 20% FCS, 100 ng/ml SCF, 50 ng/ml Flt3, 50 ng/ml TPO, 50 ng/ml IL-3, 50 ng/ml IL-6 and 100 ng/ml G-CSF for 3 days. Vital primary AML cells were then seeded onto 6-well plates (as described for Kasumi-1 cells) and challenged with antimitotic agents and/or proteasome inhibitors. Cell cycle kinetics were assessed after 24 hours by propidium iodide and annexin staining and analyzed using CellQuest (Becton Dickinson) (see below).

### Mitotic block release and synchronization experiments

To study proteolysis during a mitotic block and mitotic exit, U2Os cells were presynchronized in early S-phase for 18 hours using thymidine at a final concentration of 2 mM in the growth medium. Cells were washed with PBS, released in the presence of 200 ng/mL nocodazole, and cultured for another 18 hours. The mitotic cell fraction was collected by mitotic shake off, washed several times, plated in normal growth medium and collected at the indicated time points.

### Flow cytometry

Cell cycle distribution was assessed by DNA staining with propidium iodide and DNA content was measured based on fluorescence intensity using a FACS Calibur (Becton Dickinson). Measurements were done using CellQuest software (Becton Dickinson).

The extent to which cells induced apoptosis and finally died was measured using an approach facilitating the distinction between early apoptosis, late apoptosis and necrosis (Annexin V FITC Apoptosis Detection Kit, Calbiochem).

### Western blotting

Western blot analyses were performed as previously described [40] using anti-cyclin (Santa Cruz Biotechnology), anti-Cdc27 (MBL International Corporation), anti-Cdc20 (Santa Cruz Biotechnology) anti-Cdh1 (Calbiochem), anti-Actin (Sigma-Aldrich), and anti-alpha-Tubulin (Sigma-Aldrich) as primary antibodies. The horseradish-peroxidase-conjugated secondary antibodies used were anti-rabbit (Amersham Biosciences) and anti-mouse (Sigma-Aldrich). Densitometric quantification was performed using ImageJ.

### *In vitro* ubiquitination

*In vitro* ubiquitination experiments were done as described previously [26]. In essence, the APC/C was immunopurified from U2Os cells which were collected by mitotic shake-off from a mitotic block using an anti-Cdc27 antibody (Sigma-Aldrich) and Protein G-agarose. Ubiquitination reactions were performed in 40 mM Tris pH7.6, 0.5 mM DTT, 5 mM MgCl, 1 mM ATP, 1.5 μM ubiquitin aldehyde, 200 nM okadaic acid, 125 nM UBE1 (Boston Biochem), and 1 μM UbcH10 (Boston Biochem). Cyclin B was transcribed/translated *in vitro* (TNT Quick Coupled Transcription/Translation Kit, Promega) in the presence of S^35^-labelled methionine and used as a substrate.

### Live-cell imaging

CYS cells (specified above) were seeded onto 8-well microscopy chambers (Ibidi, 80826 or 80822) at 25.000 or 50.000 per well and grown for 24 or 48 hours under standard cell culture conditions (37°C, 5% CO_2_, 100% air humidity). BG 430 labeling was done as described previously [39]. In brief, cells were stained in 8-well microscopy chambers for 25 minutes in 200 μL of prewarmed phenol red-free DMEM supplemented with 10% fetal bovine serum, penicillin/streptomycin, sodium pyruvate and 1 μL BG 430 stock solution (1 mM in DMSO) (New England Biolabs), resulting in a final labeling concentration of 5 μM BG 430. After staining, the samples underwent several rounds of washing in DMEM^GFP^ medium (Evrogen) supplemented with 10% fetal bovine serum and L-glutamine. Image acquisition was performed using an Olympus IX-81 inverse microscope with climate chamber, using a UPLSAPO 20× objective (N.A. 0.75) and the Cell^R (v.3.3) or Scan^R Acquisition softwares (v.2.2.09). The filter set used for the analysis of BG 430 was the CFP/YFP dual bandpass filter set from Olympus. Picture acquisition was repeated every 6 or 8 minutes under standard climate chamber conditions (37°C, 5% CO_2_, 60% air humidity). Read-outs were performed using the Scan^R Analysis software (v.1.2.0.6) allowing the export of image stacks from a desired cell as well as the specific mean fluorescence intensity values.

Quantification of slow degradation in the presence of different doses of bortezomib was done in clone 11 cells (described elsewhere [21]). Pulse chase labeling of cyclin B-SNAP with TMR Star and measurement of fluorescence intensity was done as described previously [21]. During analysis (picture acquisition every 4 minutes) the cells were exposed to a combination of an agent which blocks mitosis (nocodazole, vincristine, volasertib (BI6727)) and a proteasome inhibitor (MG-132 in a nocodazole block or bortezomib in a mitotic block induced by vincristine or volasertib).

Myeloblastic Kasumi-1 cells were seeded onto 8-well microscope chambers that were coated with fibrinogen (Ibidi, 80823) at a concentration of 30,000 and 60,000 cells per well 24 hours prior to start of the imaging experiment.

### Xenotransplantation experiments

NOD/Shi-scid/IL-2Rγnull) (NOG) mice were obtained from Taconic, Denmark and kept under micro-isolators in barrier conditions. At 6–8 weeks of age, the mice were injected intravenously with 1 × 10^6^ Kasumi-1 and Molm-13 cells (Kasumi-1 cells were a gift from Michael Lübbert; Molm-13 cells were purchased from DSMZ, Braunschweig). Treatment was started 29 days after tumor cell injection. Engraftment of human hematopoietic cells in murine peripheral blood (PB) was determined prior to treatment start by flow cytometry. Only mice with > 5% of human leukemic cells in their PB were allocated to the different treatment groups. Randomization was performed on the first day of treatment. Before their allocation to an experimental group, animals were weighed and infiltration of human leukemic cells in the murine blood was determined as described above. Based on the body weight and the infiltration rate the animals were stratified into the different groups aiming for a comparable median group body weight and percentage of human leukemic cells. The investigator was not blinded to the group allocation during the experiment nor when assessing the outcome. Overall survival was used as a read-out for tumor load and antitumoral activity. All studies were approved by the regional Committee on the Ethics of Animal Experiments (G-12/86).

### Statistical analysis

For analysis of *in vivo* experiments student's t-test, two-tailed, 1-way ANOVA and log rank test were used to calculate all reported p-values. Descriptive analyses were assessed whenever appropriate and graphs (Kaplan Meier plots) were obtained using GraphPrism software (www.graphpad.com).

For analysis of live-cell imaging data, raw data of TMR Star fluorescence intensities were exported to Microsoft Excel 2002 for further calculation. For assessment of degradation slopes during a mitotic arrest, raw fluorescence intensity data from three representative mitoses were exported and the mean fluorescence intensity values were calculated. Raw data were normalized to maximum fluorescence intensity. Statistical significance was tested using a two-tailed student's *t*-test and statistical significance was assumed in case of *p*-values < 0.05.

For statistical analysis of flow cytometry data the mean +/− standard deviation were calculated from the values derived from three independent experiments. Data are either shown as a difference in percentage compared to control cells or as absolute percentages. Statistical significance was tested using a two-tailed student's *t*-test and statistical significance was assumed for *p*-values < 0.05.

## SUPPLEMENTARY MATERIALS FIGURES AND TABLES


