# Supplementary material for: Proteasome inhibition enhances the efficacy of volasertib-induced mitotic arrest in AML in vitro and prolongs survival in vivo
Source: Oncotarget. 2017 Feb 18;8(13):21153–66. doi: 10.18632/oncotarget.15503 (PMC5400573; doi:10.18632/oncotarget.15503)
Supplement: Supplementary file 1 [file oncotarget-08-21153-s001.pdf]

Proteasome inhibition enhances the efficacy of volasertib-induced mitotic arrest in AML *in vitro* and prolongs survival *in vivo*

Supplementary Materials

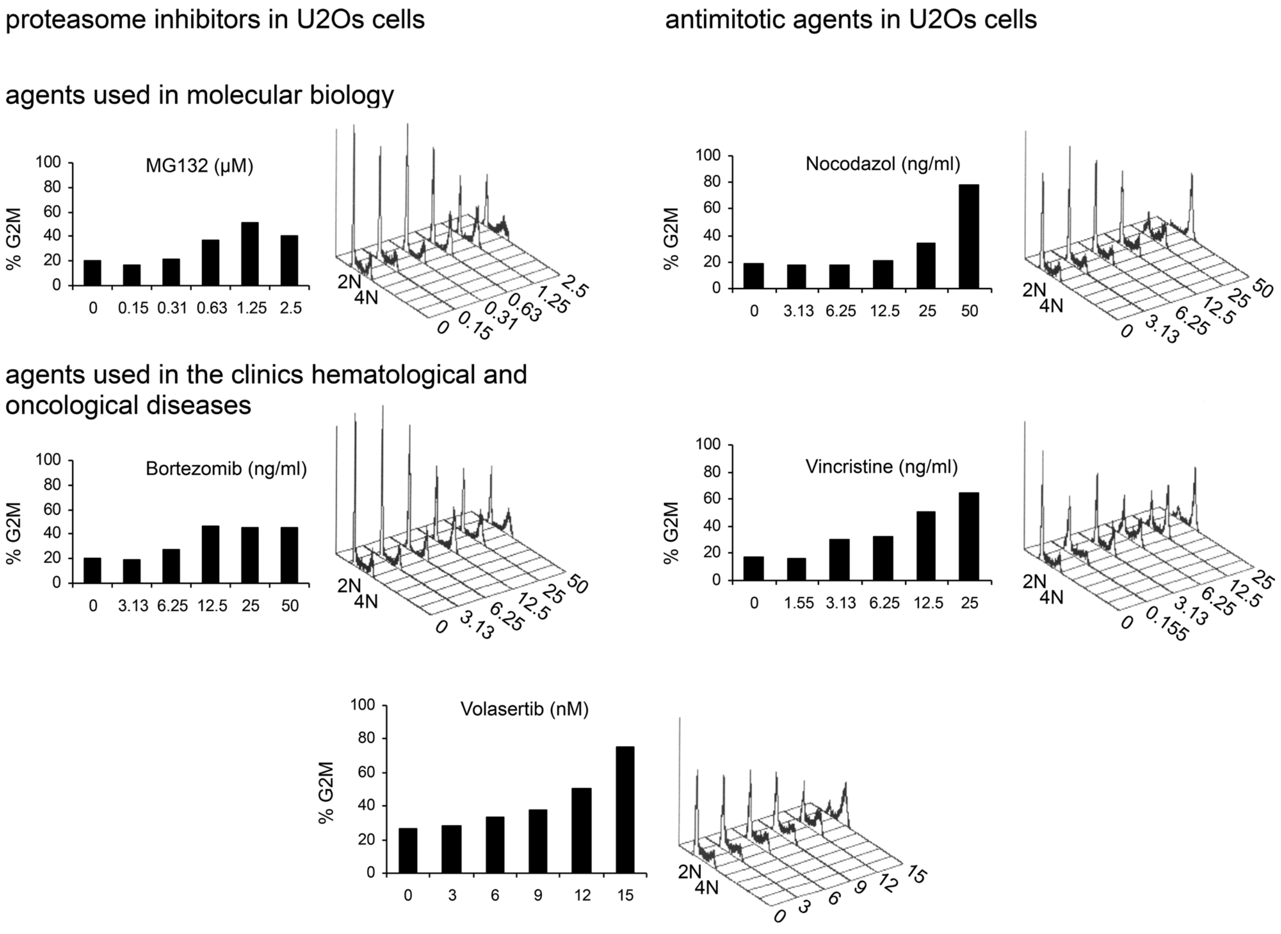

Supplementary Figure 1: Analysis of cell cycle distribution in the presence of proteasome inhibitors and antimitotic agents. U2Os cells were treated with increasing doses of proteasome inhibitors (MG132 and bortezomib) and antimitotic drugs (nocodazole, vincristine and volasertib). Cell cycle profiles for the tested drug concentrations are shown to the right. Percentages of cells in G2M were derived from the shown cell cycle profiles and are indicated in the histograms to the left.

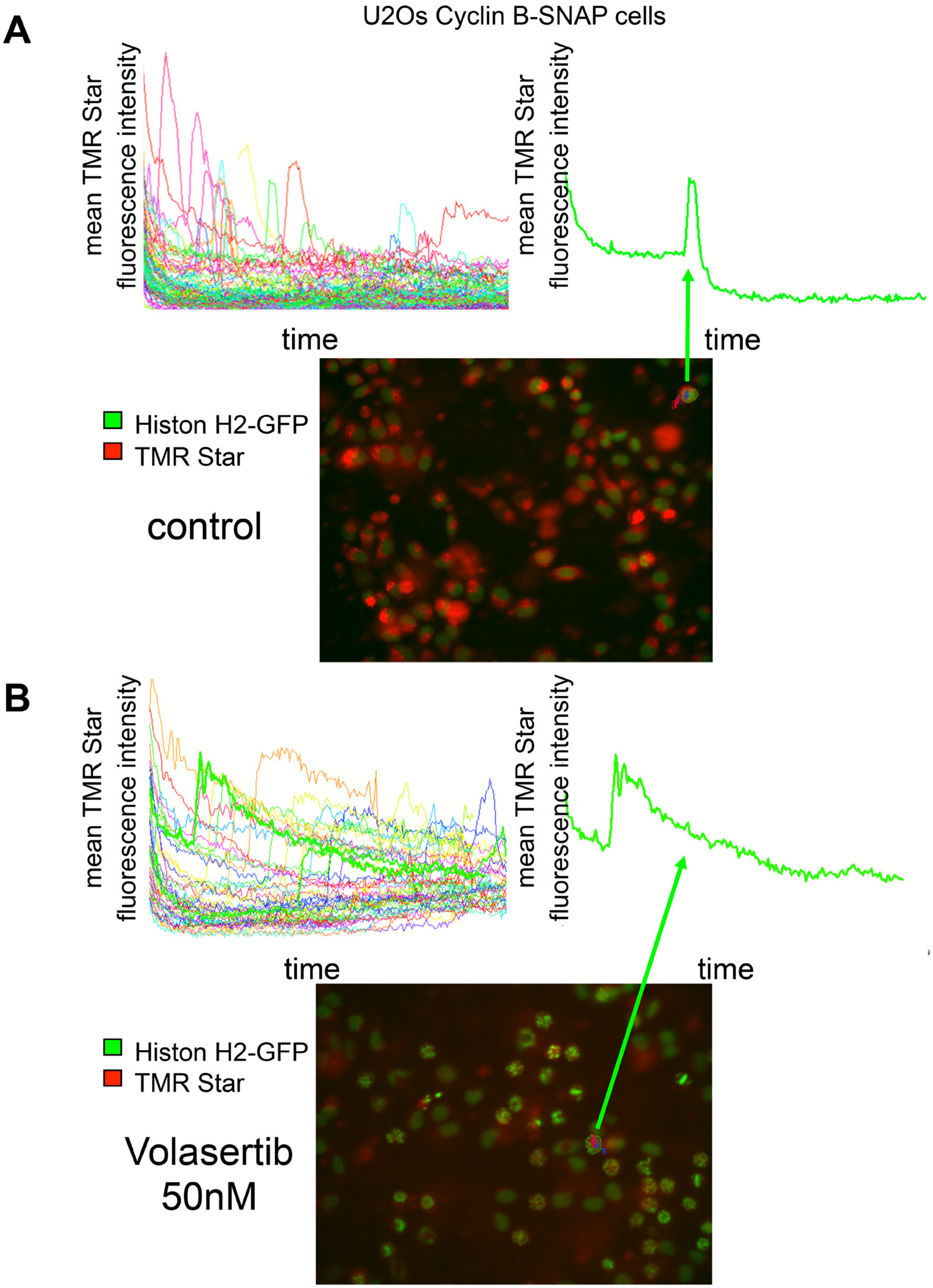

Supplementary Figure 2: Cyclin B degradation curves in the absence and presence of volasertib. Scan^R Analysis software outputs of Cyclin B-SNAP fluorescence intensity curves of representative positions/windows are shown in the upper left. The corresponding fluorescence microscopy image is shown below. The arrow points from a specific cell of interest to the corresponding degradation curve (to the upper right). (**A**) A representative position derived from a measurement of unperturbed clone 11 cells is shown. (**B**) A representative position derived from measurements of clone 11 cells treated with 50 nM volasertib is shown.

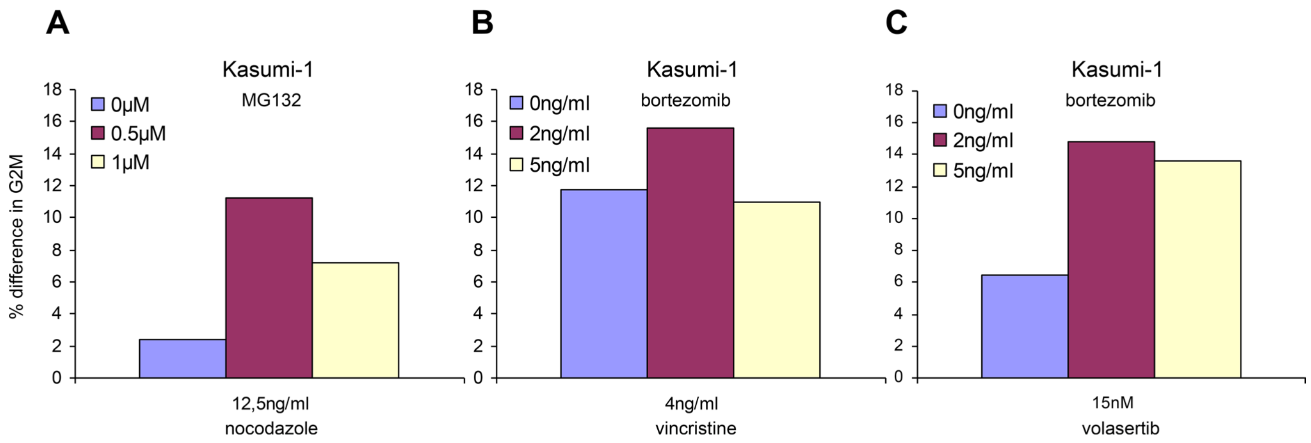

Supplementary Figure 3: Assessment of a mitotic block in the presence of ascending doses of proteasome inhibitors. Kasumi-1 cells were probed with ascending doses of the proteasome inhibitors MG132 and bortezomib and simultaneously challenged with antimitotic agents (nocodazole, vincristine and volasertib). The extent to which Kasumi-1 cells underwent a mitotic block was assessed by flow cytometry. The histograms show the response to treatment as indicated by % difference in G2M.

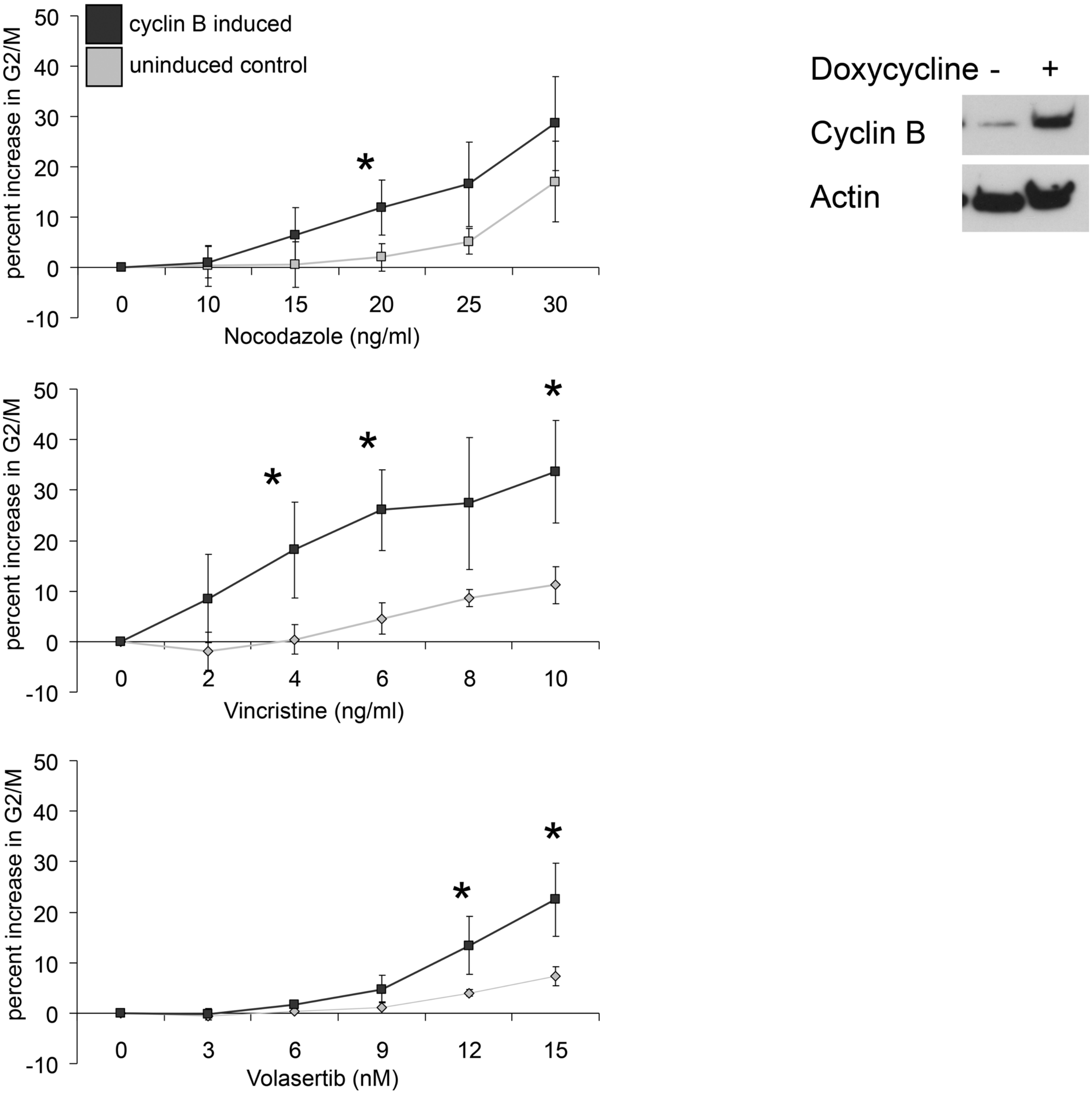

Supplementary Figure 4: Inducible overexpression of cyclin B enhances a mitotic block in Kasumi-1 cells. Cyclin B-inducible Kasumi-1 cells, capable of inducible overexpression of cyclin B upon exposition to doxycycline, were treated with different doses of antimitotic drugs (nocodazole, vincristine and volasertib). Western Blot to the upper right shows the cyclin B expression level before and after induction of cyclin B overexpression upon doxycycline. Diagrams indicating the percentage of cells in G2M in the presence of antimitotic drugs are shown to the left. Note that Kasumi-1 cells overexpressing cyclin B show a more pronounced accumulation of cells in G2M already at lower drug concentrations. Statistical significance was tested by two-tailed student's *t*-test and statistical significance was assumed for $p < 0.05$. * indicates $p < 0.05$ at the indicated time point.

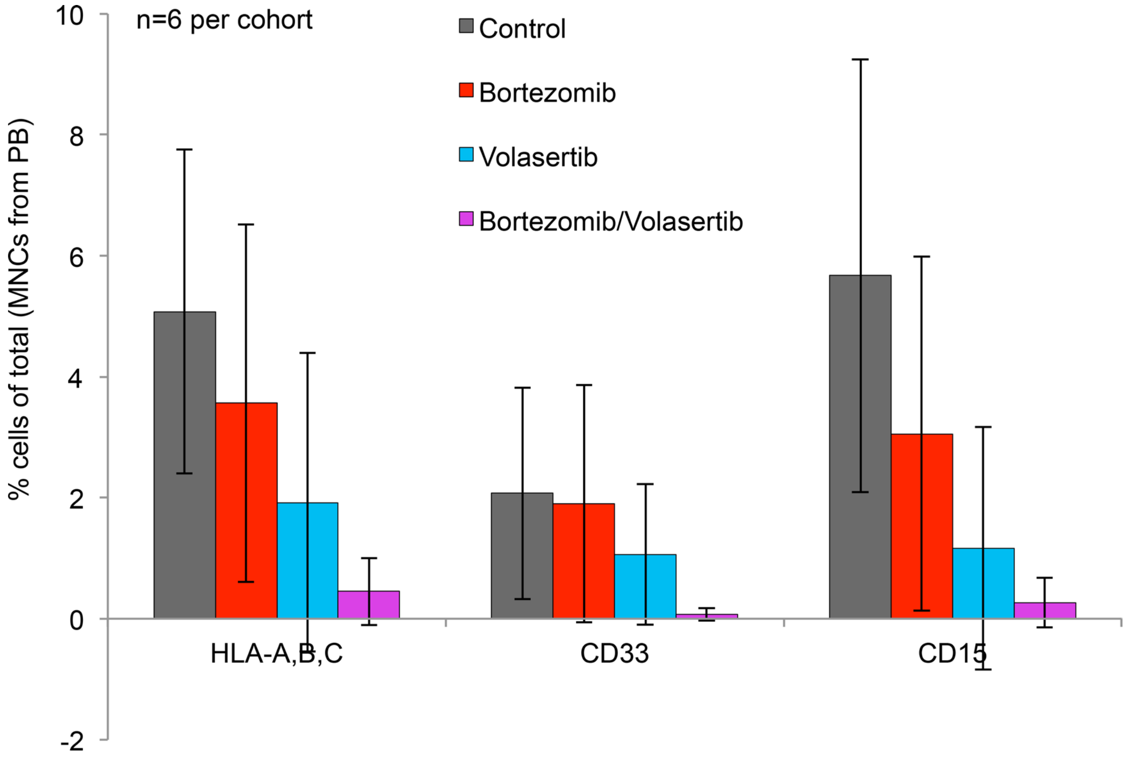

Supplementary Figure 5: The combination of bortezomib and volasertib leads to superior disease control in a Molm-13-based xenotransplant model of human AML. Combined administration of bortezomib and volasertib leads to a more prominent suppression of overt leukemia as compared to administration of volasertib alone in a Molm13-based xenotransplant mouse model. The histogram shows the percentages of HLA-A/B/C-, CD33- and CD15-positive cells in mouse peripheral blood.
